# SpliceFinder: ab initio prediction of splice sites using convolutional neural network

**DOI:** 10.1186/s12859-019-3306-3

**Published:** 2019-12-27

**Authors:** Ruohan Wang, Zishuai Wang, Jianping Wang, Shuaicheng Li

**Affiliations:** Department of Computer Science, City University of Hong Kong, 83 Tat Chee Ave, Kowloon Tong, Hong Kong, China

**Keywords:** Canonical and non-canonical splice sites, Splice site prediction, Convolutional neural network

## Abstract

**Background:**

Identifying splice sites is a necessary step to analyze the location and structure of genes. Two dinucleotides, GT and AG, are highly frequent on splice sites, and many other patterns are also on splice sites with important biological functions. Meanwhile, the dinucleotides occur frequently at the sequences without splice sites, which makes the prediction prone to generate false positives. Most existing tools select all the sequences with the two dimers and then focus on distinguishing the true splice sites from those pseudo ones. Such an approach will lead to a decrease in false positives; however, it will result in non-canonical splice sites missing.

**Result:**

We have designed SpliceFinder based on convolutional neural network (CNN) to predict splice sites. To achieve the *ab initio* prediction, we used human genomic data to train our neural network. An iterative approach is adopted to reconstruct the dataset, which tackles the data unbalance problem and forces the model to learn more features of splice sites. The proposed CNN obtains the classification accuracy of 90.25*%*, which is 10% higher than the existing algorithms. The method outperforms other existing methods in terms of area under receiver operating characteristics (AUC), recall, precision, and F1 score. Furthermore, SpliceFinder can find the exact position of splice sites on long genomic sequences with a sliding window. Compared with other state-of-the-art splice site prediction tools, SpliceFinder generates results in about half lower false positive while keeping recall higher than 0.8. Also, SpliceFinder captures the non-canonical splice sites. In addition, SpliceFinder performs well on the genomic sequences of *Drosophila melanogaster*, *Mus musculus*, *Rattus*, and *Danio rerio* without retraining.

**Conclusion:**

Based on CNN, we have proposed a new *ab initio* splice site prediction tool, SpliceFinder, which generates less false positives and can detect non-canonical splice sites. Additionally, SpliceFinder is transferable to other species without retraining. The source code and additional materials are available at https://gitlab.deepomics.org/wangruohan/SpliceFinder.

## Background

### Introduction

In recent years, high-throughput sequencing technologies have generated a large volume of genome sequences, which poses both opportunities and challenges to the identification of gene structure in genomes. The analysis of gene structure becomes one of the essential tasks in bioinformatics. A complete gene structure annotation includes the start codons, splice sites which are the boundaries between exons and introns, and stop codons. Many *in silico* methods are proposed to identify the aforementioned functional sites [1]. The success of an annotation system relies on accurate prediction of each component. In this work, we focus on the prediction of splice sites where accurate localization of splice sites can substantially help explore the structure of genes [2]. Furthermore, accurate prediction of splice sites can setup the boundaries of exons which is critical in alternative splicing prediction.

There are two types of splice sites, *donor* sites and *acceptor* sites where *donor* sites are located at the junction of exon-intron and *acceptor* sites mark the intron-exon boundaries. Two highly conserved dinucleotides are observed on the splice sites, GT for *donor* sites and AG for *acceptor* sites [3, 4]. The splice sites confirming the GT-AG consensus are called canonical splice sites.

We now introduce the main factors which affect the accuracy of splice site prediction. Firstly, the existence of the dinucleotide GT or AG is not necessary for identifying the splice sites, some non-canonical splice sites without the dimers may be observed [5–9]. Though non-canonical splice sites may not appear frequently [10, 11], some of them are vital in immunoglobulin gene expression and other important biological events [11]. Secondly, the existence of the dinucleotide GT or AG is not a sufficient condition for splice sites since dimers frequently occur at the sequences that are not splice sites. In this paper, we address the aforementioned two issues in splice site prediction.

### Related work

The existing splice site prediction tools work on data from either RNA sequences or DNA sequences. For RNA-seq based tools, TopHat [12], SpliceMap [13], and MapSplice [14] apply the alignment-based approach by mapping the reads from RNA-seq experiments to the reference genome and discovering the exon-exon junctions. The alignment-based approach makes it easier to avoid false positives since it relies on the junction signals, instead of patterns. However, the need for a reference genome limits its application. Recently, deep neural networks have been employed to predict splice sites from arbitrary pre-mRNA transcript sequences [15].

For tools based on DNA sequences, they utilized learning models to learn the features around splice sites. With more advanced machine learning algorithms designed, complex patterns are likely to be learned, which leads to the improvement in prediction accuracy. For example, GeneSplicer applies the decision tree algorithm and enhances it with Markov models to capture additional information around splice sites [16]. SpliceMachine employs linear support vector machines to build a linear model, in order to predict splice sites from high-dimensional local context representations [17]. Support vector machines with weighted degree kernel have also been applied to genome-wide predictions of splice sites [18]. In recent years, deep networks have been widely utilized to detect splice signals from genomic data. A novel deep belief network with restricted Boltzmann machines training method has been proposed for the class-imbalanced problem [19] in splice site prediction. Long short-term memory (LSTM) [20] and convolutional neural networks (CNN) [21] have also been tried to improve the performance. However, the learning models have the shortcoming of excessive false positives. To solve the problem, most tools firstly choose all the sequences with canonical signals (GT for *donor* sites and AG for *acceptor* sites) as candidate splice sites and then distinguish between true splice sites and pseudo splice sites [16–18, 21]. In spite of the decrease of false positives, these tools would miss all the non-canonical splice sites.

Based on the existing problems of splice site prediction, we propose to design a splice site prediction tool, named SpliceFinder, which has the following strengths:

(i) The model is trained with genomic data directly, so it can achieve the *ab initio* prediction of splice sites.
(ii) Not only canonical but also non-canonical splice sites can be predicted correctly.
(iii) The number of false positives decreased since SpliceFinder considers more information besides AG or GT pattern to identify splice sites.

## Methods

### Datasets

DNA sequence data (FASTA files) and annotations of the corresponding sequences (GTF files) were downloaded from Ensembl [22]. Our models were trained using human reference genome (*GRCh38*). Since the reverse strand is the reverse-complementary strand of the forward strand, we only considered the forward strand. The hg38 dataset contains 29742 genes with 21 exons per gene on average, most of these exons have duplicates due to alternative splicing [23]. We randomly chose a certain number of exons to generate training set for *donor* sites and *acceptor* sites.

For the purpose of testing the models on other species, we also downloaded the genomic sequences of *Drosophila melanogaster* (*BDGP6*), *Mus musculus* (*GRCm38*), *Rattus* (*Rnor_6.0*), and Danio rerio (*GRCz11*) from Ensembl.

### Convolutional neural network

**Fig. 1:**
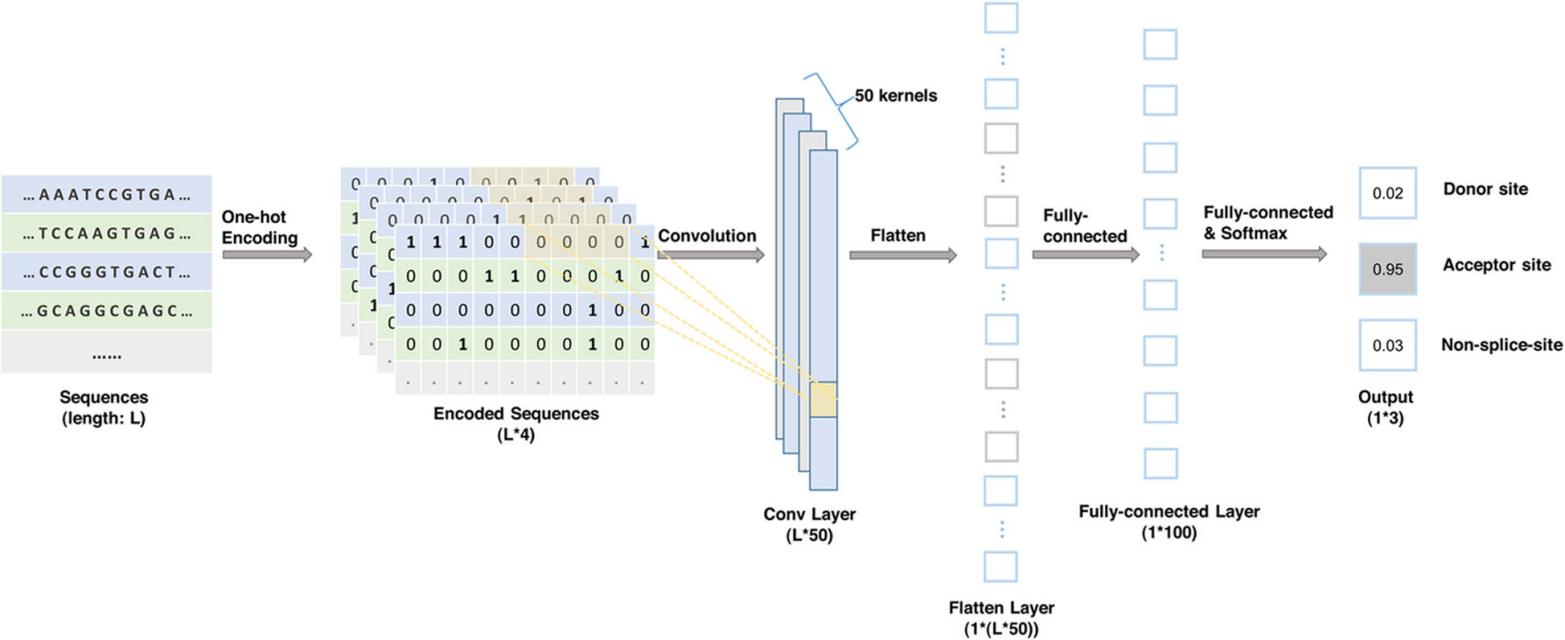
The architecture of our proposed CNN. The input of the neural network is the encoded DNA sequence with the length of L. The first layer is a 1-D convolutional layer, consists of 50 kernels with the size of 9. The second layer is a fully connected layer with 100 neurons, followed by a dropout layer. Another fully connected layer and softmax activation function are applied for the final prediction

Neural networks (NNs) consist of connections between neurons, NNs learn from dataset by adjusting the weights of the connections. However, the weights for different positions are independent, NN is not enough for finding the particular patterns of splice sites over the sequences [21]. Therefore, a convolutional layer, which enables shared weights, is added to the NN [24]. Figure 1 and the following descriptions provide a summary of the input and architecture of our CNN.

Before training, the sequence data are transformed using one-hot encoding. A (Adenine) is encoded as (1 0 0 0), C (Cytosine) is encoded as (0 1 0 0), G (Guanine) is encoded as (0 0 1 0), T (Thymine) is encoded as (0 0 0 1), and N (uncertain nucleic acid) is encoded as (0 0 0 0). As a result, each sequence can be represented with a *L*×4 matrix where *L* is the length of the sequence and 4 is the size of nucleotides vocabulary. The encoded sequence is the input of our neural network.

The first layer of our neural network is a convolutional layer. The genomic sequence is considered as a 1-D sequence window with a fixed length *L* and four channels (A, C, G, T). The convolutional layer is supposed to extract the pattern information with 50 kernels of size 9, and the length of scanning step is set to 1, for the purpose of preserving the integrity of genetic code. The output of the convolutional layer is a *L*×50 feature map, where *L* is the length of the sequence. Different numbers of convolutional layers were tried and the results are shown in Additional file 1. The NN with one convolutional layer gives the best performance. Consequently, the following experiments will apply one convolutional layer.

The following layer is a fully connected layer with 100 neurons. The fully connected layer is employed in order to improve the nonlinear expression ability of our neural network, so that the model is more likely to detect those non-canonical splicing signals. ReLU [25] is applied as the activation function in this layer. In order to avoid overfitting, a dropout layer [26] is used to randomly mask out 30% of the output. The final fully connected layer has three neurons which correspond to *acceptor* site, *donor* site, and non-splice-site. Softmax activation function [27] is used for the neurons in the last fully connected layer to convert the output into normalized probability.

For training, cross-entropy [28] is used as the loss function, and Adam algorithm [29], with the learning rate of 10^−4^, is applied for optimization. The number of epochs for training is set to 40, with the batch size of 50. We used Keras Python package to build our CNN.

### Training and testing procedure

The human reference genome (*GRCh38*) was used to construct the dataset. We obtained the location of exons from the annotation file, took sequences centered at the right and left boundaries of exons, which correspond to *donor* sites and *acceptor* sites, as the positive set of our training data, and then we took sequences centered at the intermediate position of two adjacent splicing sites as the initial negative set. The dataset contains 10000 sequences of *donor* site, *acceptor* site, and non-splice-site, which are randomly selected. Among the 30000 sequences, we used 90% for training and 10% for testing, and then 20% of the training data were set as validation data, which were used for checking network structure, hyper-parameters, and sequence length.

Next, we used our trained model to predict the splice sites on real-world long genomic sequences. Now that the model requires fixed length input, we decided to use a sliding window to detect every location on the long sequences. Only when the splicing site locates in the middle of an input sequence will our model give a positive classification result. Therefore, by moving the sliding window along the sequence and putting every subsequence into our model, we will get the exact positions of every splicing site. To decrease the number of false positives, we also used two classifiers for those subsequences considered to have splice sites. The structures of these two classifiers are the same as the structure of our proposed CNN, except that the last fully connected layer has only 2 neurons, to predict whether it is a *donor* (*acceptor*) site or false positive.

### Dataset reconstruction

In spite of the high classification accuracy, we found that when we apply the model to a long real-world sequence, most non-canonical splice sites could not be found out, and there were still many false positives. We believe the major cause is that the training set is not comprehensive. To solve the unbalance problem of the dataset, firstly, we reconstructed the positive set by achieving a canonical: non-canonical ratio of 10:1 (totally 22000 sequences). Then, for the negative set, there are too many sequences containing GT or AG that are not annotated as splice sites, so we used an iterative approach [30] to reconstruct the negative set:

(i) We used 90% of the current dataset as the training set. The model was trained with the training set. The rest of the dataset was used to test the performance of our CNN on the current dataset.
(ii) The model was used to predict splice sites on the sequence of a randomly chosen human gene with a sliding window.
(iii) The false positives generated from the last step were added to the negative set. The new negative set, together with the positive set, constituted the new dataset.

**Fig. 2:**
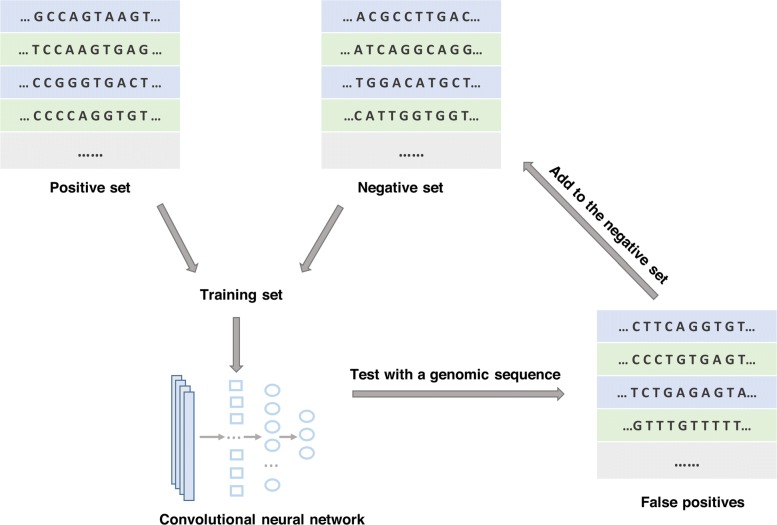
The iterative approach for negative set reconstruction. At each iteration, the trained CNN is tested with a randomly chosen genomic sequence, the false positives are collected and added to the training data, which will be used to train our CNN at the next iteration

This procedure was repeated until the number of false positives did not decrease anymore when testing a gene sequence which is set aside in advance. These steps are illustrated in Fig. 2. The method forces the convolution neural network to learn more features for the classification task.

### Performance evaluation

Our models can be applied to discriminating short sequences and predicting splice sites on long sequences. For short sequences classification, we measured performance in terms of accuracy, area under receiver operating characteristics curve (AUC), recall, precision, and F1 score:

$$Recall = \frac{TP}{TP+FN},$$

$$Precision = \frac{TP}{TP+FP},$$

$$F1\;score = (\frac{Recall^{-1}+Precision^{-1}}{2})^{-1}.$$

Since all the measures except accuracy are applicable for two-class classification, and other methods used for comparison are designed only for two-class problems, we calculated AUC, recall, precision, and F1 score for *donor* site and *acceptor* site separately. The ROC curves and precision-recall curves were also made for the performance evaluation.

For long genomic sequences, on the one hand, most of the existing tools can not find all the splice sites no matter how to set the parameters; on the other hand, the prediction with low recall is meaningless even though the number of false positives is small. In order to consider both the value of recall and the number of false positives, we counted the number of false positives when 100% and 80% of the splice sites are successfully predicted separately.

## Results

### Testing different length of input

**Fig. 3:**
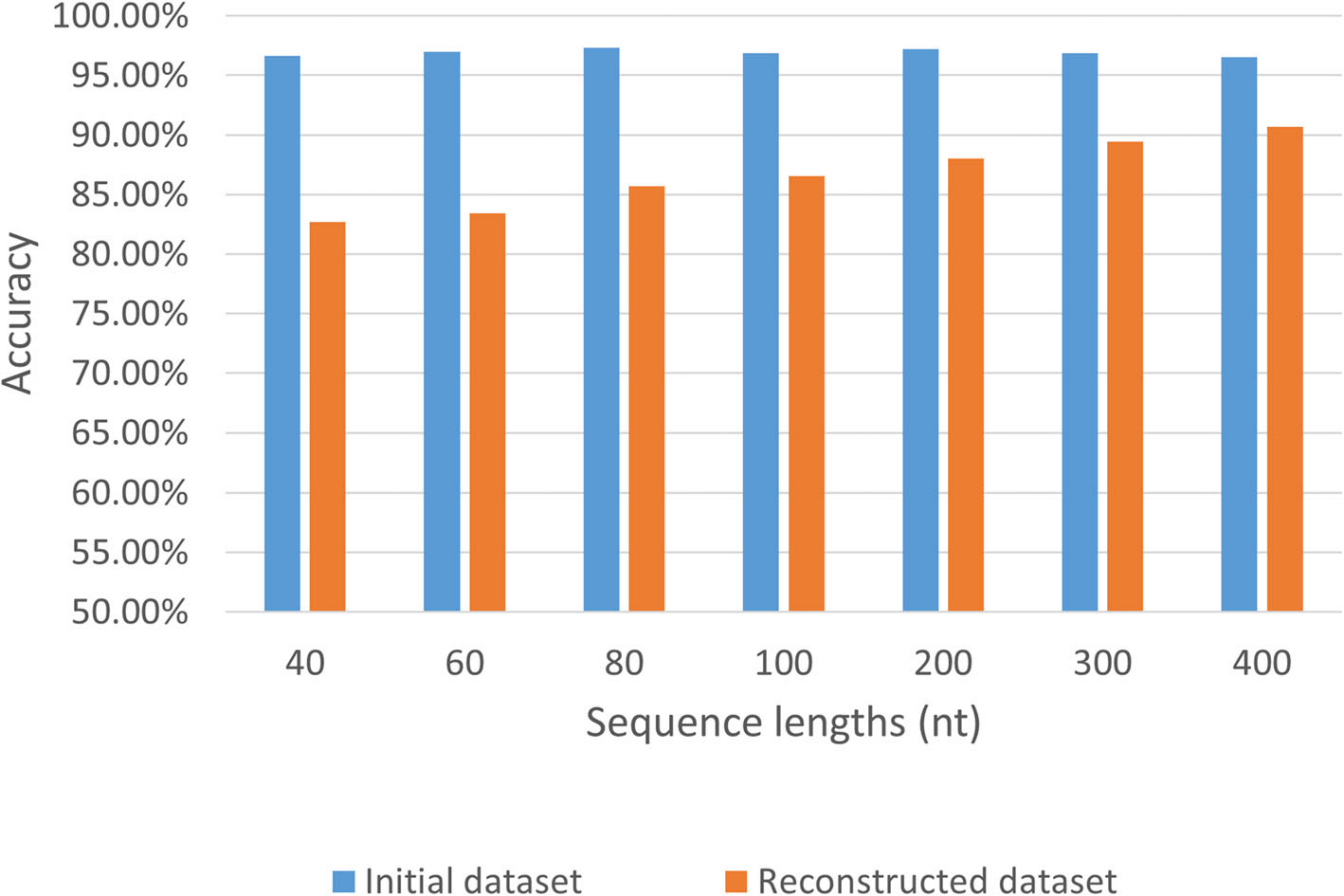
The effect of sequence length on accuracy. Varying the sequence lengths from 40 to 400 nt, the classification accuracies for the test set of initial dataset and reconstructed dataset are compared

To choose the most suitable region for training, we used sequences of different lengths as the input of our models. Since the sequence lengths used in other splice site prediction tools range from 40 to 400 nt, we varied the input lengths in this range. Using the initial dataset, we found that all the test accuracies reach 95%. However, the accuracies have significant declines after the iterative process because of the increased complexity of test data. For instance, the accuracy decreases from 96.9% to 83.2% for sequence length of 40 nt, and for sequence length of 400 nt, the accuracy changes from 96.5% to 90.3%. Longer sequences help models keep good performance since they provide more information. As shown in Fig. 3, CNN achieves the best performance with the input length of 400 nt for the reconstructed dataset. Accordingly, subsequent experiments will use this length of input.

### Decomposing the output of CNN

**Fig. 4:**
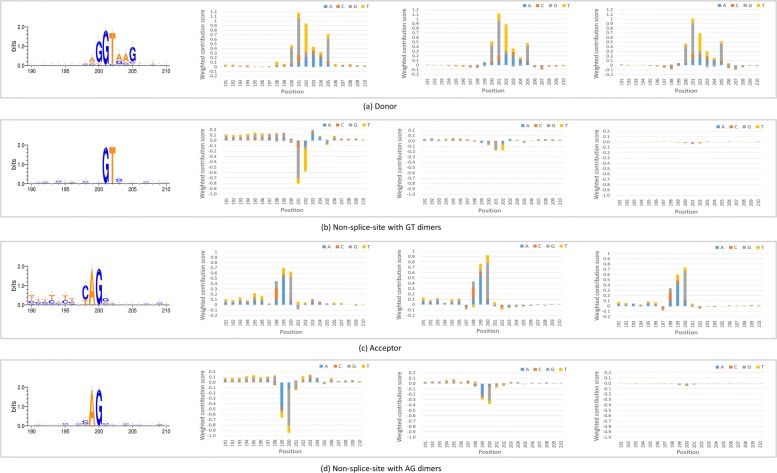
The sequence logos and average weighted contribution scores of nucleotides near the splice site. For *donor* sites, *acceptor* sites, and non-splice-sites with canonical signals, the average weighted contribution scores of different models for each nucleotide near the splice site (located at the position between 200 and 201) is shown. From left to right, the models are generated from the 1st, 50th, and 100th iteration. The sequence logos are made [32] to show the difference of patterns between true and false splice sites. **a** Donor. **b** Non-splice-site with GT dimers. **c** Acceptor. **d** Non-splice-site with AG dimers

To improve the interpretability of our neural network, we utilized DeepLIFT to analyze the contributions of different regions inside the sequence window to the output. DeepLIFT uses the backpropagation method to decompose the prediction of neural network on the input and computes the weighted contribution scores for every part of the input, thus making neural network no longer a “black box” [31]. We randomly chose 100 sequences with *donor* sites, 100 sequences with *acceptor* sites, 100 non-splice-site sequences with GT dimers, and 100 non-splice-site sequences with AG dimers, from human reference genome, and then applied three models generated from different periods of the iterative process to classifying these sequences. The average weighted contribution scores for 20 nucleotides near the splice sites are computed, which show the influence of each nucleotide on the right decision. A positive score means a positive role in making the right decision while a negative score means an opposite effect. The result is shown in Fig. 4. For sequences with *donor* site or *acceptor* site, the contribution scores of GT or AG are high for all of the three models. However, for non-splice-site sequences with the dinucleotide GT or AG, the negative influence of the two dimers decreases a lot for the models generated from later periods of the iterative process.

### Comparison of classification performance

We compared our CNN with common machine learning algorithms (Logistic regression [33], Decision tree [34], Random forest [35], SVM [36] with linear and RBF kernel), DBN [37], and LSTM [38], the last two algorithms have been applied to predicting splice junctions previously and all the parameters are set as described by the authors [19, 20]. We trained and tested the mentioned algorithms using the reconstructed dataset. There are also tools that require more information besides genomic sequences, like RNA sequences, or only accept long sequences as inputs. For the second case, we will compare these tools with our models on long genomic sequences later.

**Fig. 5:**
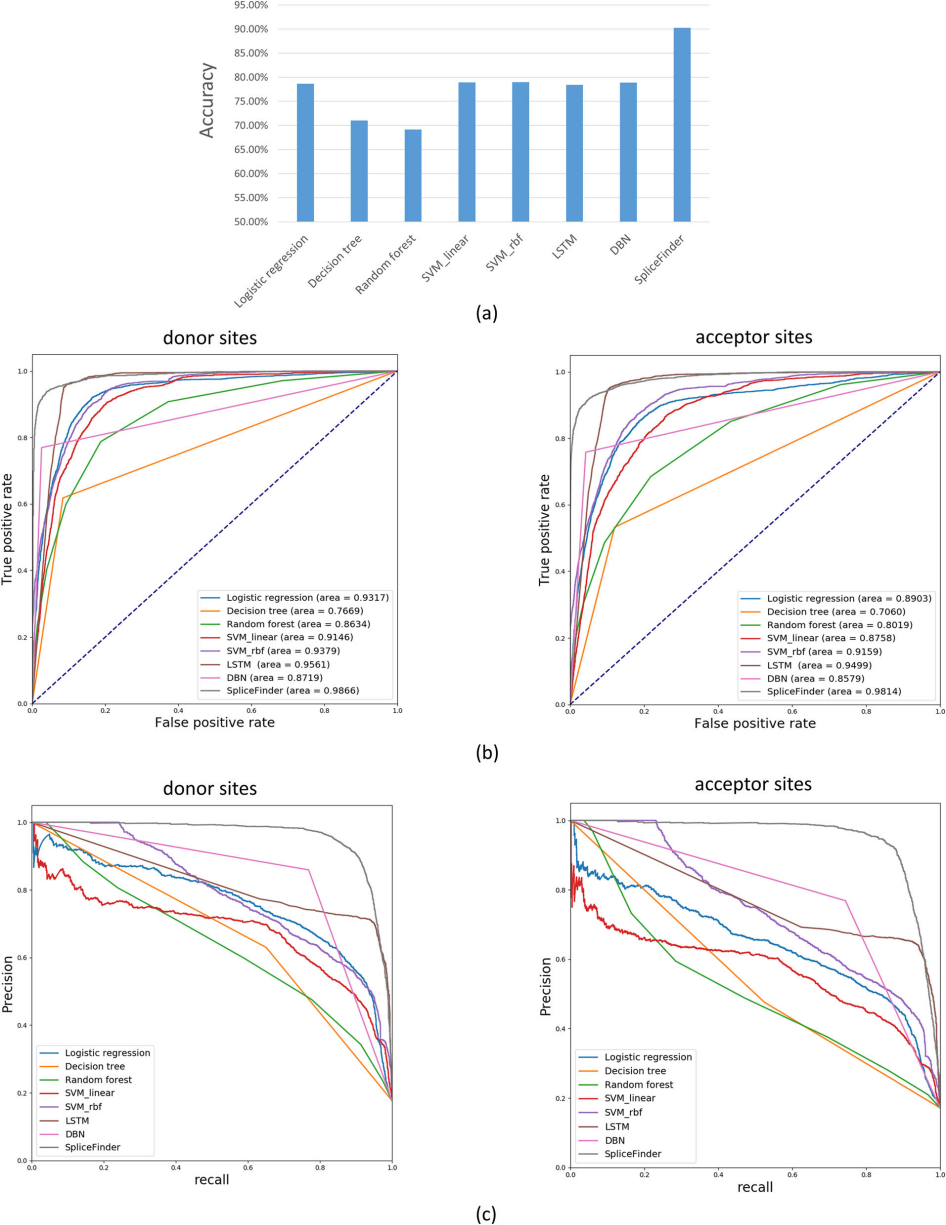
Comparison of classification performance of different methods on the test set of the reconstructed dataset. The compared measures include (**a**) classification accuracy; (**b**) ROC curve for *donor* sites (left) and *acceptor* sites (right); (**c**) Precision-recall curve for *donor* sites (left) and *acceptor* sites (right)

Compared with other algorithms, SpliceFinder has the best performance with regard to all the measures. Figure 5 (a) presents the comparison of accuracies between SpliceFinder and other machine learning algorithms. The classification accuracy of SpliceFinder exceeds 90% while the accuracies of all other machine learning algorithms do not reach 80%. Figure 5 (b) and (c) show the ROC curves and precision-recall curves for *donor* site and *acceptor* site respectively. SpliceFinder achieves the largest areas under the curves apparently. Additionally, in terms of recall, precision, and F1 score, SpliceFinder obtains higher scores than other algorithms, the comparison result can be found in Additional file 2.

### Prediction performance on genomic sequences

To evaluate our method and compare it with other tools, we used SpliceFinder to predict the splice sites on three randomly chosen human genomic sequences. These sequences are set aside in the iterative process.

**Fig. 6:**
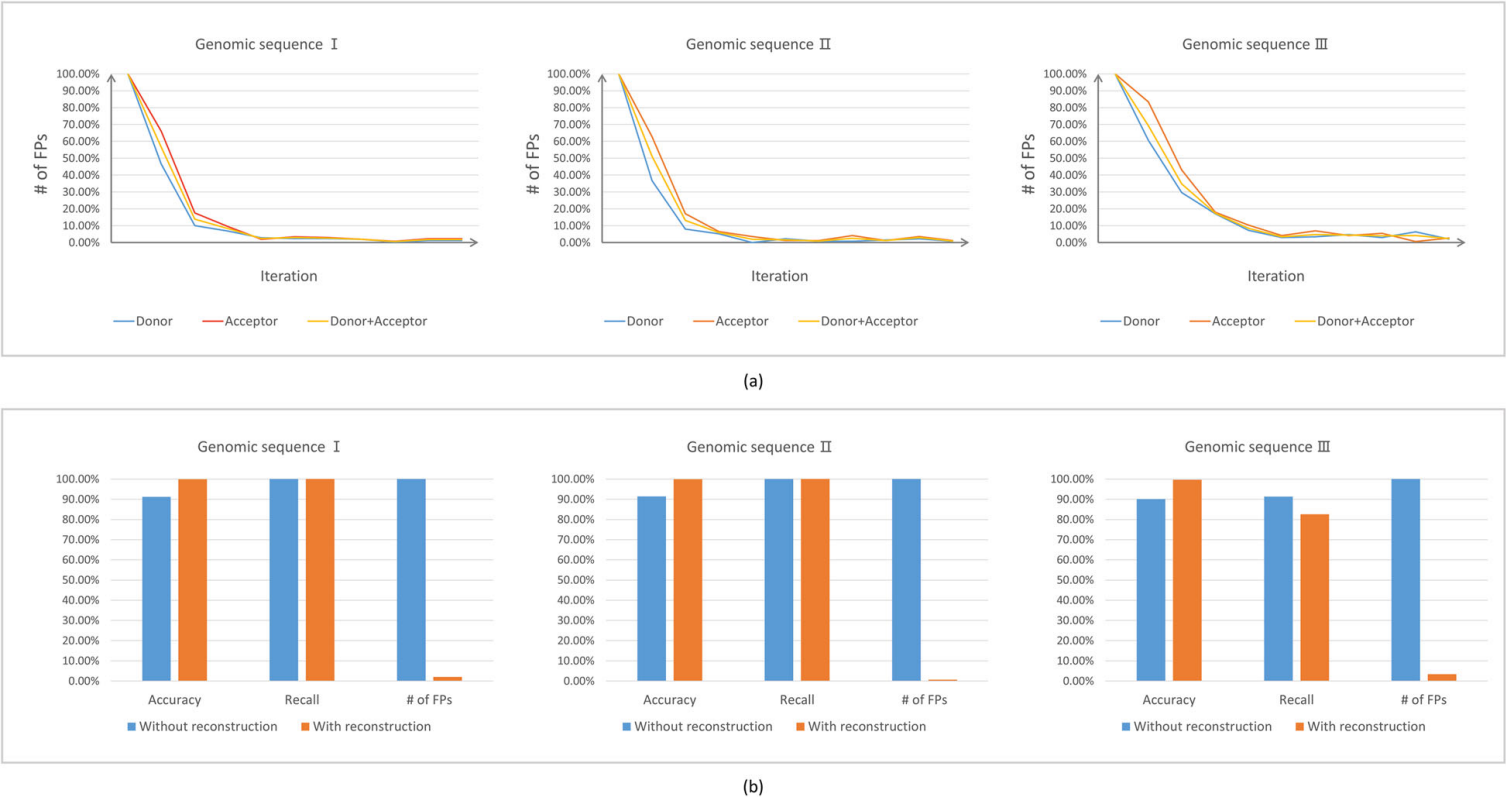
The prediction performance improves after dataset reconstruction. **a** Using the models generated in the iterative process to predict the splice sites on three randomly chosen genomic sequences, false positive numbers of both *donor* site and *acceptor* site are shown. The false positive numbers of the initial model are set as 100%. **b** The comparison of accuracy, recall, and false positives numbers between models with and without dataset reconstruction

**Fig. 7:**
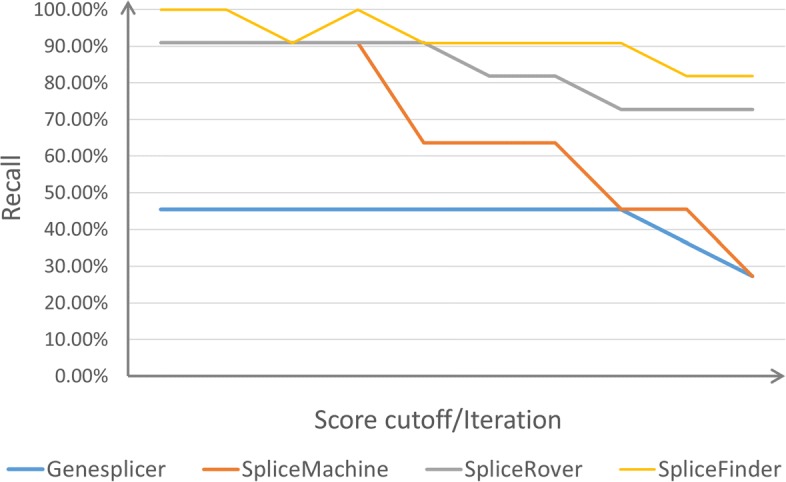
Comparison of recall of different softwares for *donor* sites of Genomic Sequence III. Using different score cutoff or models generated in the iterative process, the recall values of the four softwares, for *donor* sites of Genomic Sequence III, are calculated

We employed the models generated in the iterative process and found the numbers of false positives have sharp declines with iteration, shown in Fig. 6 (a). Since the numbers of false positives decrease and the values of recall remain high, the model obtains better accuracies on the three genomic sequences after dataset reconstruction (Fig. 6 (b)). Next, we compared the performance of SpliceFinder with GeneSplicer [16], SpliceMachine [17], and SpliceRover [21]. When recall is 1, the number of false positives was calculated. Since some tools can not reach the recall of 1 at all, we also compared the number of false positives when recall is over 0.8. As shown in Table 1, with recall reaching 1 and 0.8, SpliceFinder generated the least false positives. During the experiment, we noticed that when the score cutoff was set to be 0, SpliceRover would consider all the sequences with GT patterns as *donor* sites and all the sequences with AG patterns as *acceptor* sites. However, Fig. 7 shows that SpliceRover still misses a *donor* site even with the score cutoff set as 0 for Genomic Sequence III. Therefore, there is a *donor* site without GT pattern on this sequence. Despite this, for SpliceFinder, the recall of models generated from early iteration is 1, which means the models have the ability to find the non-canonical splice sites.

**Table 1:** Comparison of prediction performances of different softwares on three randomly chosen genomic sequences

(a)			
**Genomic Sequence I**	*Donor*	*Acceptor*	*Donor* & *Acceptor*
GeneSplicer	N/A	N/A	N/A
SpliceMachine	159	62	221
SpliceRover	19	16	35
SpliceFinder	7	5	**12**
**Genomic Sequence II**	*Donor*	*Acceptor*	*Donor* & *Acceptor*
GeneSplicer	N/A	N/A	N/A
SpliceMachine	72	54	126
SpliceRover	3	6	9
SpliceFinder	1	2	**3**
**Genomic Sequence III**	*Donor*	*Acceptor*	*Donor* & *Acceptor*
GeneSplicer	N/A	N/A	N/A
SpliceMachine	N/A	N/A	N/A
SpliceRover	N/A	N/A	N/A
SpliceFinder	24	35	**59**
(b)			
**Genomic Sequence I**	*Donor*	*Acceptor*	*Donor* & *Acceptor*
GeneSplicer	N/A	N/A	N/A
SpliceMachine	159	62	221
SpliceRover	19	3	22
SpliceFinder	5	5	**10**
**Genomic Sequence II**	*Donor*	*Acceptor*	*Donor* & *Acceptor*
GeneSplicer	9	4	13
SpliceMachine	10	4	14
SpliceRover	0	3	**3**
SpliceFinder	1	2	**3**
**Genomic Sequence III**	*Donor*	*Acceptor*	*Donor* & *Acceptor*
GeneSplicer	N/A	N/A	N/A
SpliceMachine	20	90	110
SpliceRover	444	21	465
SpliceFinder	6	6	**12**

The numbers of false positives when recall reaches 1 (a) and 0.8 (b) are shown. (Note: N/A implies the software can not reach the recall of 1 or 0.8, no matter how to set the parameters. The best performance is in bold.)

### Testing on other species

**Fig. 8:**
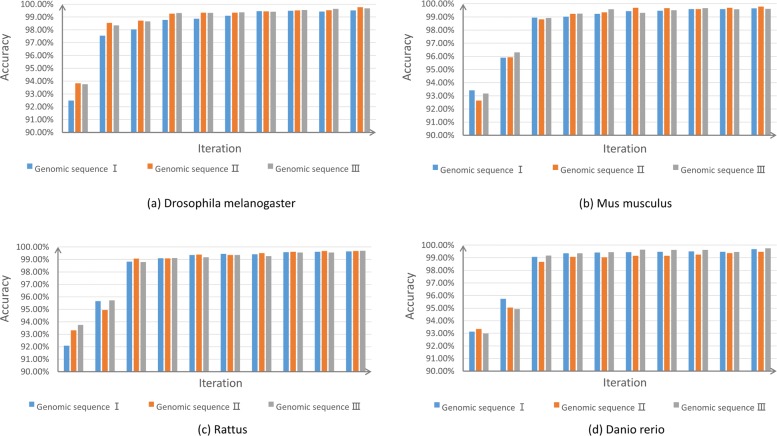
The splice site prediction accuracy of our models for other species. For (**a**) *Drosophila melanogaster*, (**b**) *Mus musculus*, (**c**) *Rattus*, and (**d**) *Danio rerio*, the models generated in the iterative process are applied to predicting the splice sites on three randomly chosen genomic sequences

**Fig. 9:**
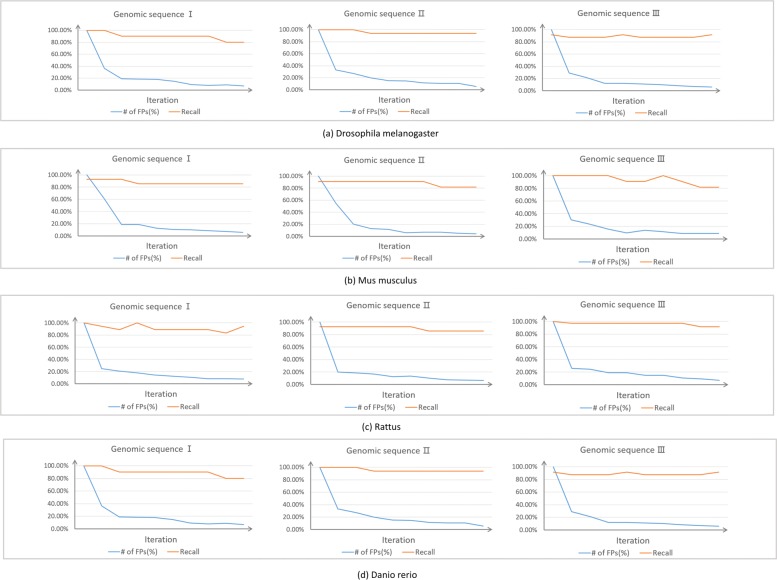
The false positive numbers and recall of our models for other species. For (**a**) *Drosophila melanogaster*, (**b**) *Mus musculus*, (**c**) *Rattus*, and (**d**) *Danio rerio*, the numbers of false positive and values of recall are calculated to show more details of the prediction performance for other species

Since GT-AG rule can be applied to the splice sites of all eukaryotic genes, we also used our trained models to predict splice sites on the genomic sequences of *Drosophila melanogaster*, *Mus musculus*, *Rattus*, and *Danio rerio*. 3 long sequences for each species were chosen randomly. Shown in Fig. 8, with iteration, the models obtained higher accuracies. Since high accuracy is not enough for our task, we also calculated the numbers of false positives and values of recall (See Fig. 9). For convenience, both *donor* site and *acceptor* site are considered as the positive set, so the recall in Fig. 9 is the percentage of splice sites to be predicted successfully. It can be seen that with iteration, the models are tended to give less false positives, but keep the recall higher than 0.8 for the 12 genomic sequences of different species in Fig. 9. Other mentioned machine learning algorithms are also be compared on other species, SpliceFinder achieves the best performance on the four species (Additional file 3). Additionally, we found the model trained with *Homo sapiens* data have the same performance as the model trained with other species data or multiple species data, on the genomes of all the four species (Additional file 4).

## Discussion

### Analyzing the data reconstruction

The initial dataset is randomly generated from human genomic sequences. In this case, only a small number of sequences without splice sites contain the GT or AG pattern, while almost all the splice sites confirm the GT-AG consensus. Using this simple feature, many algorithms have good performance in the classification tasks even with the input of short length. However, when being applied to the prediction task on long genomic sequences, we found that our models still miss those non-canonical splice sites. More seriously, any sequence with GT or AG pattern is easily misclassified as *donor* site or *acceptor* site, which leads to a large number of false positives. Most tools also have these two problems. To decrease the number of false positives, many existing splice site prediction algorithms transfer the problem to two classification problems: discriminating sequences with splice sites from sequences without splice sites but with the dinucleotide GT or AG for *donor* and *acceptor* sites separately [16–18, 21]. However, their methods focus on canonical splice sites, only sequences with the consensus GT or AG will be classified, which will definitely miss non-canonical splice sites.

Therefore, we decided to consider the problem in a different way. We think the most important cause for the missed splice sites and false positives is the unbalance of the training dataset, With the simple training set, the classifier cannot learn enough information to find non-canonical splice sites and exclude non-splice-sites with canonical signals. Increasing the proportion of non-canonical splice sites and the iterative process make the training set cover more information, therefore the models can deal with those unusual cases better after the reconstruction of dataset. Although the classification accuracy is decreased, the models have better prediction performance on long genomic sequences. Of course, since the information in the training set is much more difficult to learn, it requires longer sequences as input and models with stronger pattern-find ability.

### Strengths of SpliceFinder

Based on the experimental results, we believe SpliceFinder has the following strengths:

(i) Trained with data generated directly from human genomic sequences, SpliceFinder has achieved *ab initio* splice site prediction. The experimental results have proved that SpliceFinder has good prediction performance using only genomic sequences.
(ii) To improve the sensitivity of our models for the non-canonical splice sites, we increased the number of splice sites without GT-AG patterns in the training data, so unlike other existing tools, SpliceFinder also considers non-canonical splice sites.
(iii) Instead of simply increasing the score cutoff, we used an iterative process to reconstruct the dataset, in order to decrease the number of false positives. We can see SpliceFinder makes less false positive predictions while still successfully finding the true splice sites.
(iv) SpliceFinder is a 3-class model, which can directly give the classification result of *donor* site, *acceptor* site or non-splice-site. Compared with other tools with 2-classes models, SpliceFinder is more straightforward and convenient to use.
(v) SpliceFinder can be used to predict splice sites on the genomic sequences of other species with no need to retrain, so it can be applied to the annotation of new species.

### Future work

Our future work will continue to explore the following topics:

(i) Noticing that SpliceFinder can also be applied to other species without retraining, we plan to combine SpliceFinder with other tools based on RNA-seq [12–14], SpliceFinder provides the locations of splice sites for reference, RNA-seq based tools use the alignment approach to identify the actual splice sites. We strongly believe the two methods will work together and play complementary roles, especially for new species without reference genome.
(ii) A complete annotation system needs to predict not only the splice sites but also the transcript start sites (TSSs) and transcription termination sites (TTSs) [39]. There are also conserved sequences around TSSs and TTSs, for example, the well-known TATA box is universally observed in the core promoter region [40]. However, how to handle the promoter sequences without TATA box and the non-promoter sequences with TATA patterns remains a problem. It is also a pattern-based task, so we plan to adjust the structure of our CNN and expand the application of our tool.

## Conclusions

In this paper, we introduced a new tool for splice site prediction, named SpliceFinder. SpliceFinder applies convolutional neural network to classify sequences to *donor* site, *acceptor* site or non-splice-site. With a sliding window, it can predict the exact position of every splice site on long genomic sequences with less false positives and high recall. Compared with other splice site prediction tools, SpliceFinder has better prediction performance, it also has the ability to find the non-canonical splice sites. Additionally, the models trained with human genome are also applicable to other species without retraining, which makes SpliceFinder useful in the annotation of new species.

## Supplementary information

**Additional file 1** **Figure S1** The performance of models with different number of layers.

**Additional file 2** **Figure S2** Evaluation of different methods with various metrics.

**Additional file 3** **Figure S3** The splice site prediction accuracy of different methods for other species.

**Additional file 4** **Figure S4** The performance of models trained with data of other species.

## Data Availability

The datasets analysed during the current study are available in the Ensembl repository, https://asia.ensembl.org/info/data/ftp/index.html.

## Abbreviations

AUC: Area under receiver operating characteristics curve
CNN: Convolutional neural network
LSTM: Long short-term memory
mRNA: Messenger RNA
NN: Neural network
Pre-mRNA: Precursor-messenger RNA
ROC: Receiver operating characteristics
TSS: Transcript start site
TTS: Transcript termination site
