# Supplementary material for: SpliceFinder: ab initio prediction of splice sites using convolutional neural network
Source: BMC Bioinformatics. 2019 Dec 27;20(Suppl 23):652. doi: 10.1186/s12859-019-3306-3 (PMC6933889; doi:10.1186/s12859-019-3306-3)
Supplement: Supplementary file 1 — Additional file 1 Figure S1 The performance of models with different number of layers. [file 12859_2019_3306_MOESM1_ESM.docx]

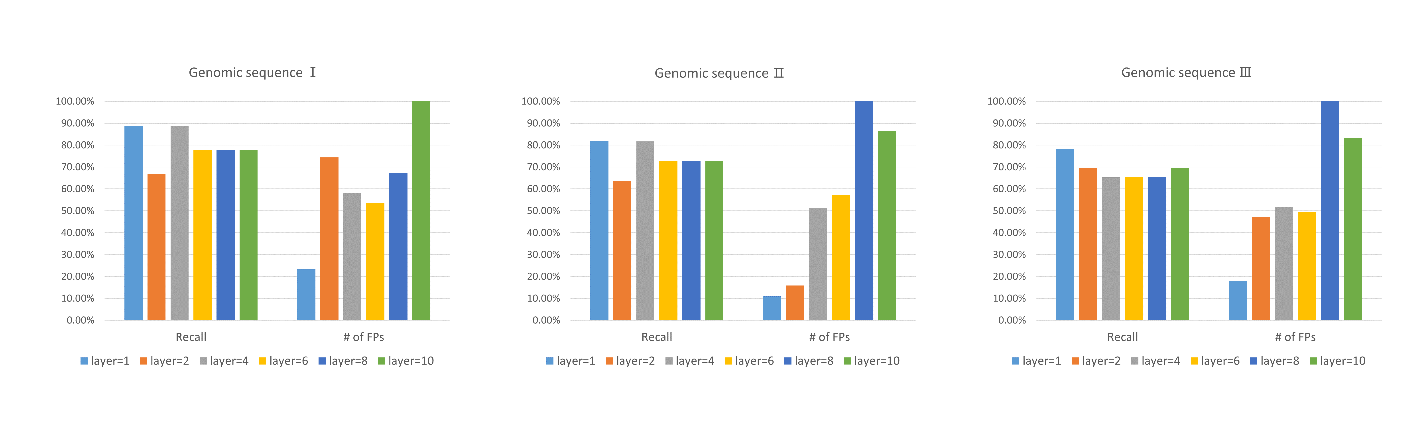


**Figure S1 The performance of models with different number of layers.** The models with different number of layers are applied to three randomly chosen genomic sequences; the value of recall and number of false positives are calculated.
