# Supplementary material for: SpliceFinder: ab initio prediction of splice sites using convolutional neural network
Source: BMC Bioinformatics. 2019 Dec 27;20(Suppl 23):652. doi: 10.1186/s12859-019-3306-3 (PMC6933889; doi:10.1186/s12859-019-3306-3)
Supplement: Supplementary file 2 — Additional file 2 Figure S2 Evaluation of different methods with various metrics. [file 12859_2019_3306_MOESM2_ESM.docx]

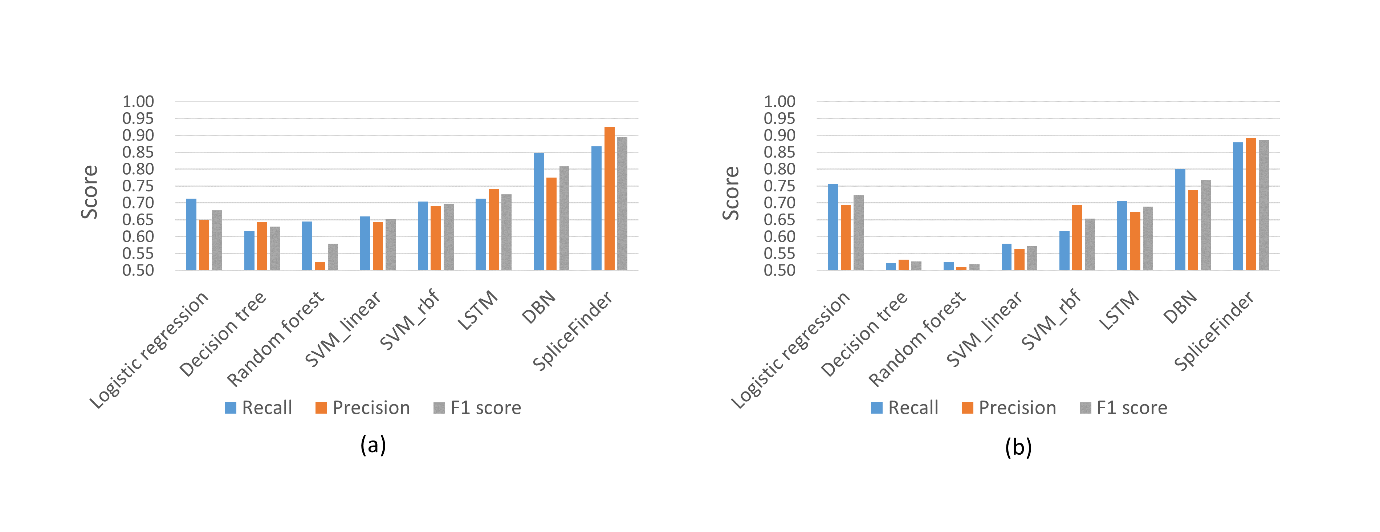


**Figure S2 Evaluation of different methods with various metrics.** Recall, precision and F1 score for (a) *donor* sites and (b) *acceptor* sites are compared among different methods respectively.
