# Supplementary material for: SpliceFinder: ab initio prediction of splice sites using convolutional neural network
Source: BMC Bioinformatics. 2019 Dec 27;20(Suppl 23):652. doi: 10.1186/s12859-019-3306-3 (PMC6933889; doi:10.1186/s12859-019-3306-3)
Supplement: Supplementary file 3 — Additional file 3 Figure S3 The splice site prediction accuracy of different methods for other species. [file 12859_2019_3306_MOESM3_ESM.docx]

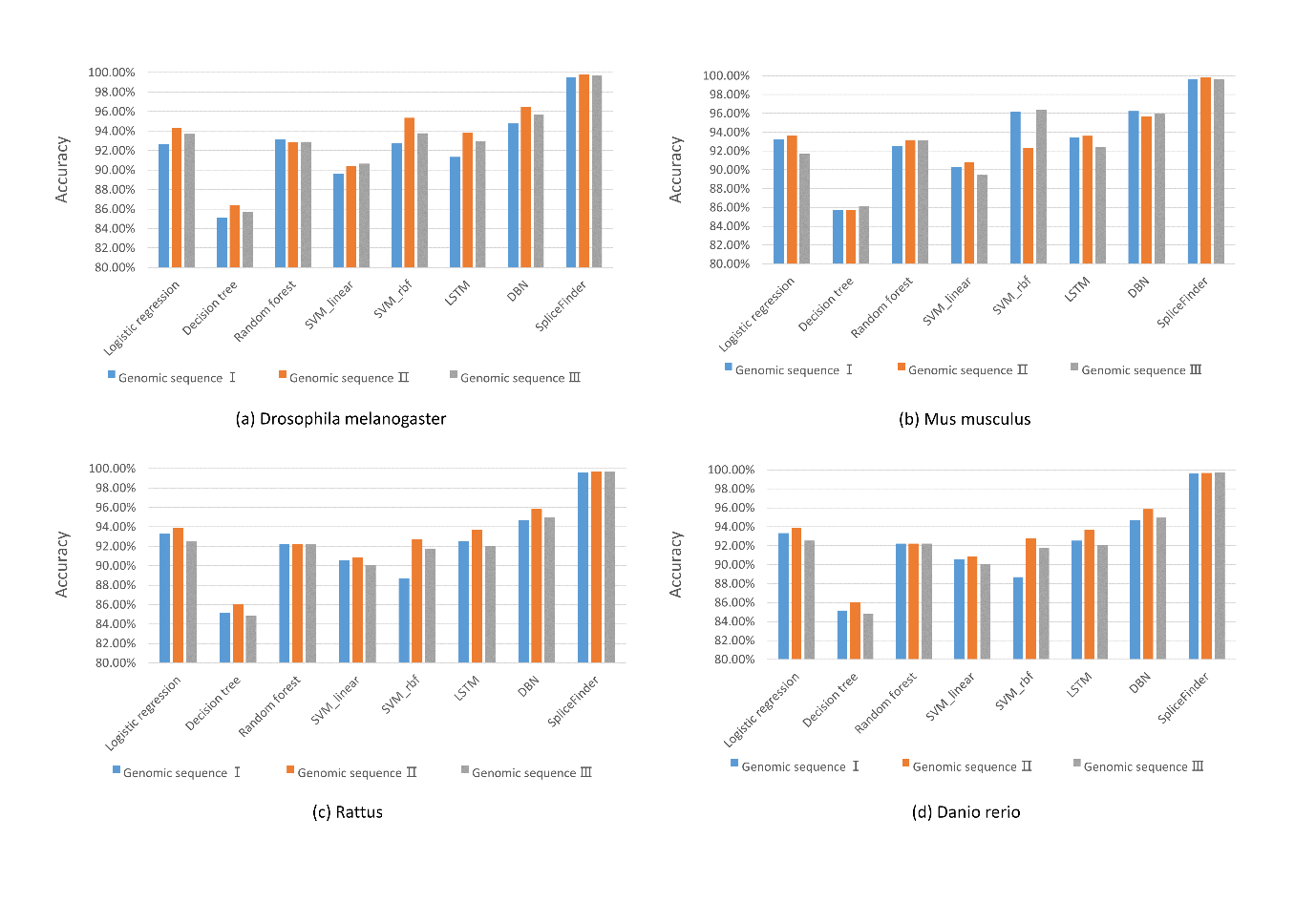


**Figure S3 The splice site prediction accuracy of different methods for other species.** For (a) *Drosophila melanogaster*, (b) *Mus musculus*, (c) *Rattus* and (d) *Danio rerio*, other methods are also applied to predicting the splice sites on three randomly chosen genomic sequences, and the prediction accuracies are compared with SpliceFinder.
