# Supplementary material for: SpliceFinder: ab initio prediction of splice sites using convolutional neural network
Source: BMC Bioinformatics. 2019 Dec 27;20(Suppl 23):652. doi: 10.1186/s12859-019-3306-3 (PMC6933889; doi:10.1186/s12859-019-3306-3)
Supplement: Supplementary file 4 — Additional file 4 Figure S4 The performance of models trained with data of other species. [file 12859_2019_3306_MOESM4_ESM.docx]

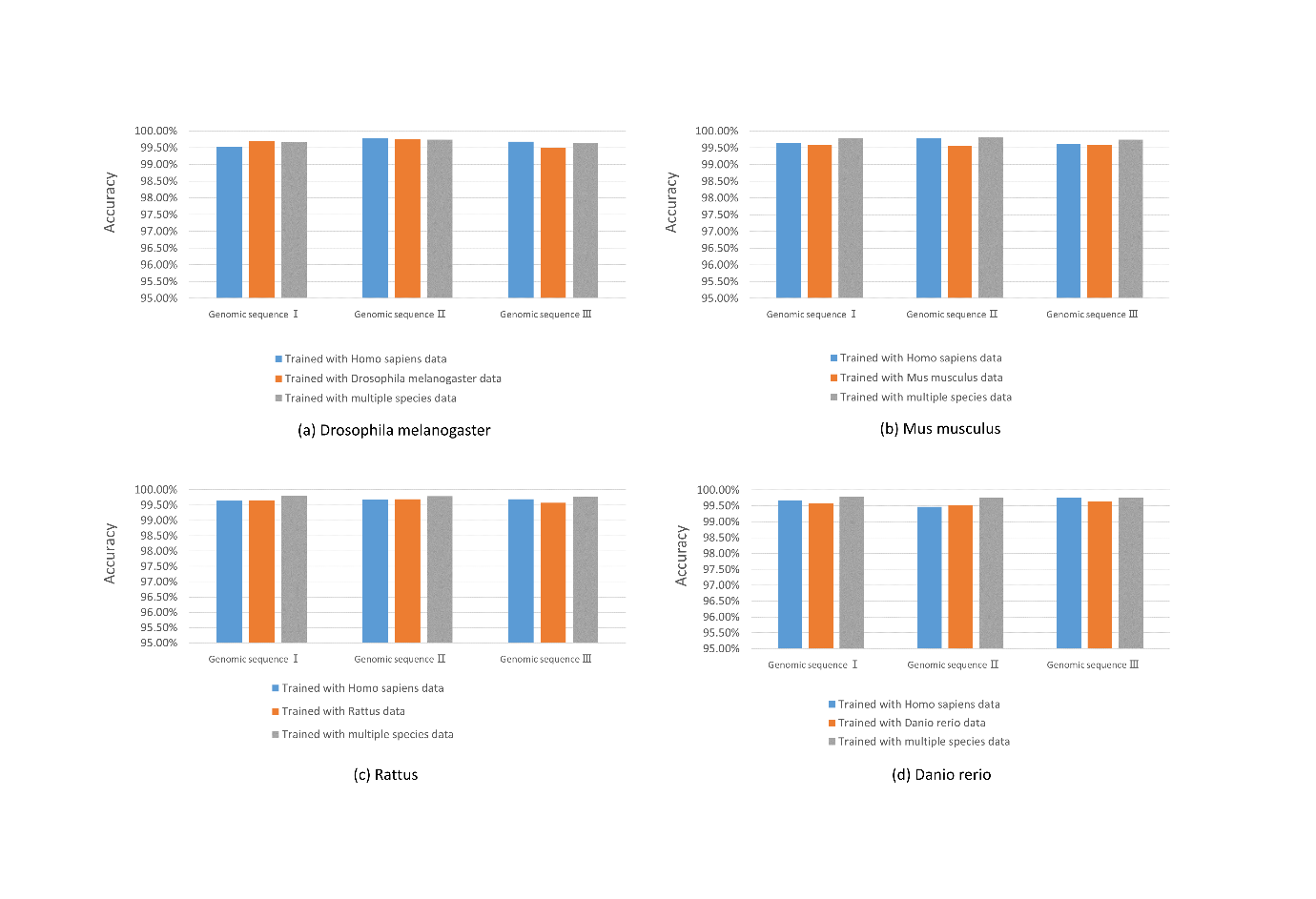


**Figure S4 The performance of models trained with data of other species.** The models trained with other species and multiple species are used to predict splice sites on three randomly chosen sequences of genes for (a) *Drosophila melanogaster*, (b) *Mus musculus*, (c) *Rattus* and (d) *Danio rerio*.
